# First direct determination of the ^93^Mo half-life

**DOI:** 10.1038/s41598-021-99253-5

**Published:** 2021-10-05

**Authors:** I. Kajan, S. Heinitz, K. Kossert, P. Sprung, R. Dressler, D. Schumann

**Affiliations:** 1 Paul Scherrer Institut (PSI), Villigen, Switzerland; 2 Belgian Nuclear Research Centre (SCK CEN), Mol, Belgium; 3 Physikalisch-Technische Bundesanstalt (PTB), Braunschweig, Germany

**Keywords:** Mass spectrometry, Nuclear chemistry, Characterization and analytical techniques, Mass spectrometry

## Abstract

This work presents the first direct measurement of the ^93^Mo half-life. The measurement is a combination of high-resolution mass spectrometry for the determination of the ^93^Mo concentration and liquid scintillation counting for determining the specific activity. A ^93^Mo sample of high purity was obtained from proton irradiated niobium by chemical separation of molybdenum with a decontamination factor larger than 1.6 × 10^14^ with respect to Nb. The half-life of ^93^Mo was deduced to be 4839(63) years, which is more than 20% longer than the currently adopted value, whereas the relative uncertainty could be reduced by a factor of 15. The probability that the ^93^Mo decays to the metastable state ^93m^Nb was determined to be 95.7(16)%. This value is a factor of 8 more precise than previous estimations. Due to the man-made production of ^93^Mo in nuclear facilities, the result leads to significantly increased precision for modelling the low-level nuclear waste composition. The presented work demonstrates the importance of chemical separations in combination with state-of-the-art analysis techniques, which are inevitable for precise and accurate determinations of nuclear decay data.

## Introduction

Although radioactivity was discovered more than 120 years ago^1^, decay properties of a considerable number of radioactive isotopes close to the line of stability are still not precisely known. Intriguingly, this knowledge gap also includes isotopes with medium or long half-lives that are readily produced within nuclear facilities or due to interactions of matter with cosmic radiation like ^32^Si or ^53^Mn^2–4^. The majority of decay data for radioisotopes are based on measurements that were conducted at the beginning of the second half of the last century. However, the development of more precise measurement tools like high resolution inductively coupled plasma—mass spectrometry (ICP-MS), accelerator mass spectrometry (AMS) as well as the improvements in the liquid scintillation counting (LSC) together with new powerful chemical separation methods have opened the possibility to improve the statistical uncertainty and accurateness of such data.

The half-life of a radionuclide can be determined by various methods. Often, when the half-life is not too long, it can be determined by means of repeat measurements. Such a method, however, is limited to radionuclides of half-lives up to ca. 100 years and crucially depends on the stability of the sample and the measurement setup. Moreover, it may require repeated measurements for a long time (e.g. several years). In such a case, a more suitable alternative is a half-life determination via a combination of precise determinations of the activity and the number of atoms of the respective radionuclide.

However, two requirements need to be met for the second method. Firstly, if the number of atoms is determined by mass spectrometry, the sample needs to be free from isobaric and polyatomic interferences. Secondly, for the activity measurements, the efficiency of the measurement setup needs to be precisely known and the sample must not contain radioisotopes that interfere with the signal of the target nuclide.

Despite the seeming simplicity of the involved techniques, the half-lives of a significant number of not so rare radioisotopes are still insufficiently constrained; sometimes because isobaric impurities hamper quantifying the number of atoms or because the pure radioisotope is hard to obtain in sufficient amounts for precise measurements. Another major complication during radionuclide production for half-life measurements is the simultaneous production of other radioisotopes of the target element which may require an additional separation using a mass separator.

**Figure 1:**
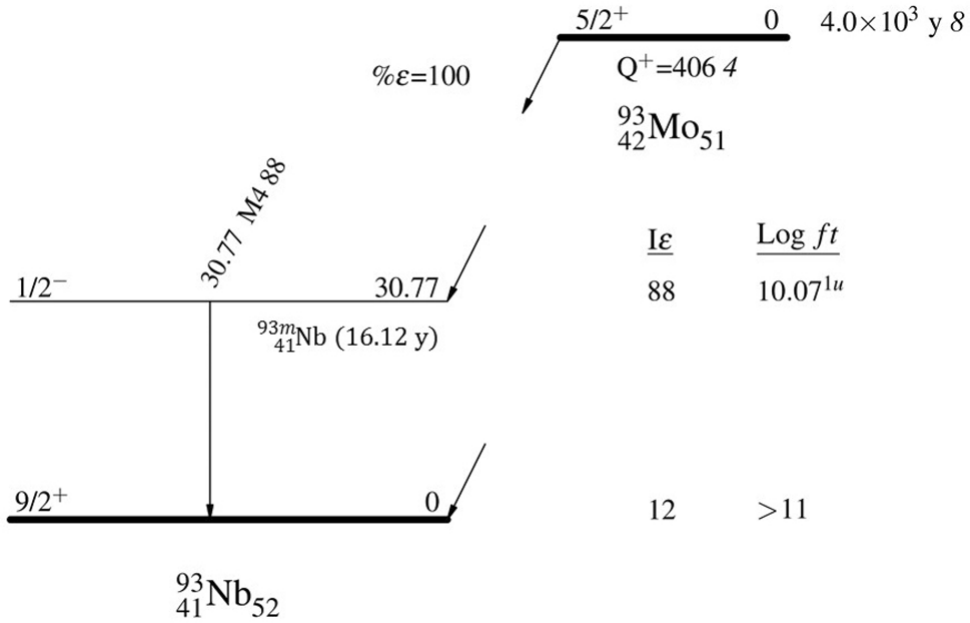
Evaluated decay scheme of ^93^Mo from^5^. Reprinted from Nuclear Data Sheets, 112/5, Coral M. Baglin, Nuclear Data Sheets for A = 93, 1163–1389, Copyright (2011), with permissionn from Elsevier.

^93^Mo is one of the last four radionuclides (the other three being ^91^Nb, ^205^Pb and ^236^Np^5–9^) with a half-life longer than 1 year and less than 100 million years whose half-life was never measured directly. ^93^Mo decays purely via electron capture (EC) to the metastable ^93m^Nb (*T*_1/2_ = 16.12(12) y) and the ground state of ^93^Nb. The decay scheme of ^93^Mo is presented in Fig. 1.

The currently accepted evaluated half-life of 4000(800) years is marked as tentative and carrying a significant uncertainty^5^. This is very surprising because it is the only isotope across the chart of nuclei produced by neutron activation of a stable isotope, where the decay properties are so vaguely known. The main source of ^93^Mo is the neutron capture of ^92^Mo in molybdenum rich materials, typically steel, Iconel alloys or cables used for constructions within nuclear power plants^10–12^. Molybdenum is also a potential material for nuclear fusion devices where high neutron fluxes unavoidably lead to the production of ^93^Mo as one of the main long-lived radioactive products^13^. Other sources of ^93^Mo in nuclear waste are particle accelerator facilities where the neutrons are emitted due to nuclear reactions. The proton accelerator at Paul Scherrer Institute where the molybdenum containing alloys are used for window materials to separate different part of the accelerator complex^14^ can serve as an example. Due to its long half-life ^93^Mo becomes the dominant radioactive dose contributor to humans in low and intermediate level radioactive waste when the shorter-lived radionuclides have decayed in the time period from 5000 to 30,000 AD^15^. Highlighting the requirement of a better-constrained half-life value of ^93^Mo, preliminary calculations show ^93^Mo to become the major radionuclide in the final repository of low-level nuclear waste over the time^16^.

First efforts in search for a long-lived Mo isotope have been undertaken at Oak Ridge National Laboratory more than 80 years ago. In these early investigations of neutron irradiated molybdenum, the ^93^Mo half-life has been estimated to be at least 70 years^17^. The first indirect determination of the half-life^18^ is based on the comparison of isotope production yields for (d,2n) reactions with a deuteron beam energy of 21 MeV in the atomic mass region of A = 85–109 with ^93^Mo produced by chemical separation from a bombarded niobium foil. The ^93^Mo activity was determined by measuring the relaxation radiation with X-ray spectrometry, while the number of produced atoms was estimated by comparing it to (d,2n) reactions on ^85^Rb, ^103^Rh and ^109^Ag, assuming a constant production yield within this atomic mass interval. The obtained value for the ^93^Mo half-life was later adjusted by the evaluator to the currently adopted value taking into account updated nuclear decay properties used in the work of^5,18^.

In this work we demonstrate the power of state of art chemical separations combined with modern instrumentation to determine the nuclear properties of radionuclides with very high precision. The isobaric impurities present the biggest obstacle in the precise determination of the number of atoms by mass spectrometry. Therefore, chemical separations play the crucial role in half-life determination by an absolute activity measurement. We communicate here chemical separation yielding significantly high decontamination factors of ^93^Mo from the interfering ^93^Nb isobar leading to precise determination of the ^93^Mo half-life by absolute activity determination.

## Experimental techniques and methodology

### Separation and purification of ^93^Mo sample

In this work, we produced a sample of around 5 × 10^15^ atoms of ^93^Mo from a 2.1 mm thick high purity niobium disc which was irradiated with 72 MeV protons. The cross section of the ^93^Nb(p,n)^93^Mo reaction is approximately 15 mb^19^. The content of radionuclides in the irradiated disc was investigated by the means of gamma-ray and X-ray spectroscopy. Three parts of the irradiated niobium disc with high radioactivity content identified by means of autoradiography were cut out. A more detailed description of the used Nb degrader, its processing and characterization is given in the Supplementary materials. These were dissolved in a mixture of 2 mL 48% HF and a 100 µL 70% HNO_3_. The separation of ^93^Mo from niobium and radioactive contaminants consisted of two separate chemical systems. In the first one the acid composition was adjusted to 6.5 mol/L HCl/0.1 mol/L HF and applied to the column filled with 2 mL of tributylphosphate (TBP) resin. Under this chemical composition molybdenum is fully retained on resin whereas niobium together with ^22^Na, ^60^Co, ^93^Zr and ^54^Mn contaminants pass through the chromatographic column. The fraction containing ^93^Mo was thereafter eluted from the resin by distilled water from a MilliQ system. In order to determine the decontamination factor (*D*_F_) from niobium, the sample was spiked before the separation with carrier free ^95^Nb obtained from the inhouse produced ^95^Zr/^95^Nb generator (for details see Supplementary material). The decontamination factor is defined as:

$${D}_{\mathrm{F}}=\frac{{C}_{{\mathrm{Nb}}_{\mathrm{L}}}}{{C}_{{\mathrm{Nb}}_{\mathrm{E}}}}\equiv \frac{{A}_{{95}_{{\mathrm{Nb}}_{\mathrm{L}}}}}{{A}_{{95}_{{\mathrm{Nb}}_{\mathrm{E}}}}}$$

where *C*_Nb_ represents the concentration of niobium in the sample in the loading (L) and elution (E) fractions of the separation, that is corresponding to the measured specific radioactivity of ^95^Nb (${A}_{{95}_{\mathrm{Nb}}}$) in the loading and elution fractions corrected for the decay during the separation procedure and measurement. Hence, the decontamination factor from niobium was determined by γ-spectrometric measurements using the 765.8 keV line of ^95^Nb measured by an n-type HPGe detector. The measurements were performed with a live time of ca. 24 h and corrected for the decay of ^95^Nb during this period.

**Figure 2:**
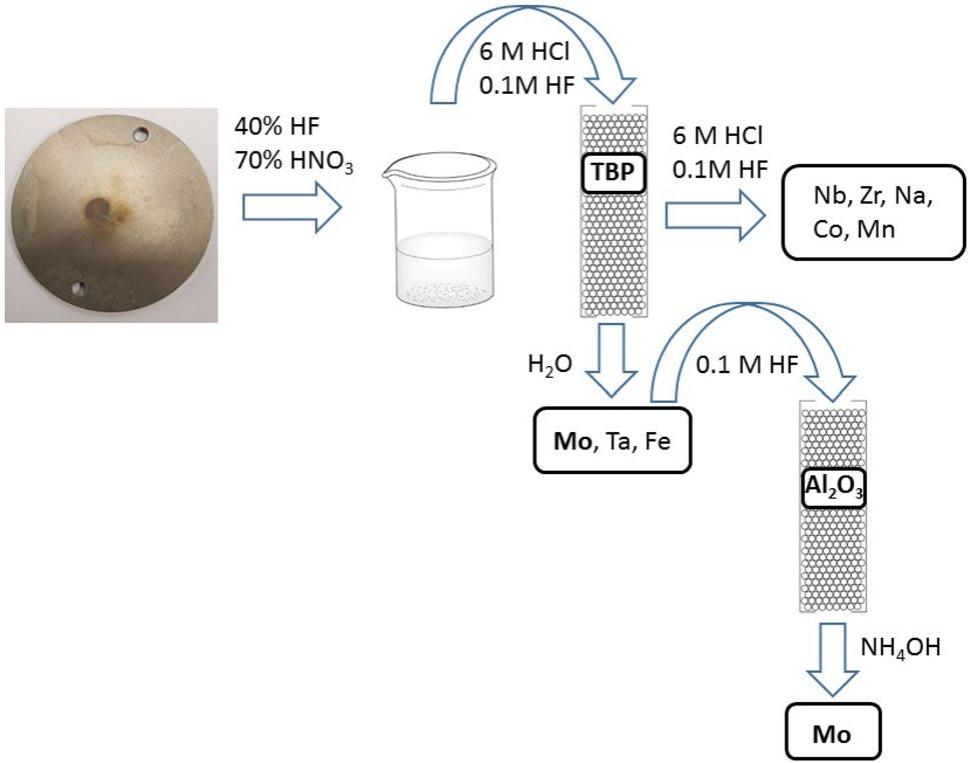
Schematics of the separation procedure for ^93^Mo master solution.

The second cycle of purification was performed on alumina substrate in a chromatographic column according to^20^. The eluate from the TBP resin was evaporated to dryness, re-dissolved in 0.1 mol/L HF and spiked again with ^95^Nb tracer. The solution was thereafter pushed through a chromatographic column filled with alumina substrate. During this procedure Zr, Nb, Mo, Ta and Fe were retained on the alumina substrate. The fraction containing ^93^Mo was thereafter selectively eluted from the substrate by 20% NH_4_OH solution. During this step, a nearly complete separation from remaining niobium as well as from traces of iron and ^179^Ta was achieved. The simplified schematics of the separation procedure is shown in Fig. 2.

Each of the three sub-samples from the irradiated Nb disc underwent a separation procedure on both columns separately before they were merged together and underwent one more separation on TBP resin resulting in the “^93^Mo master solution”.

### Mass spectrometric analysis

The homogeneous ^93^Mo master solution was split to use one part for LSC measurements and the other part for MC-ICP-MS determinations of the ^93^Mo content using a Nu Instruments Plasma 3 MC-ICP-MS. Given the large first ionization potential of Argon^21^ the inductively coupled Ar-plasma ion source of this mass spectrometer efficiently ionizes almost all elements of the periodic table. This efficient ionization capability and the ability of the state-of-the-art Plasma 3 MC-ICP-MS to detect up to 22 ion beams simultaneously allow highly precise determinations of intensity-ratios of mass-specific ion beams of the same or multiple elements. Exploiting this ability two different methods were employed to define the ^93^Mo content of the master solution: gravimetric standard addition with an extra addition of Zr as internal standard^22^ as well as reverse isotope dilution^23^. The former method makes use of the increase in the intensities of ^95^Mo and ^97^Mo ion beams per gravimetrically defined addition of natural Mo (LGC Molybdenum Standard 976(3) µg/g, *k* = 2, LOT # 987189-15, traceable to NIST SRM 314, lot #130418), cancelling intensity variations due to plasma fluctuations by referencing to ion beams of ^90^Zr or ^91^Zr from a gravimetrically defined addition of natural Zr (LGC Zirconium Standard 993(2) µg/g, *k* = 2, LOT # 150521R-15, traceable to NIST SRM 3169, lot #130920). The latter method—in the following referred to as “reverse isotope dilution”—utilizes the change in ^93^Mo-to-^95,97^Mo ion beam intensity ratios per gravimetrically defined addition of natural Mo.

All solutions were introduced into the system via a self-aspiring “PFA-ST MicroFlow” consuming ca. 50 μL/min and an “Apex HF” desolvating nebulizer (both from Elemental Scientific). Ion beams from mass 90 (Zr) through to 97 (Mo) were collected simultaneously in Faraday cups connected to amplifiers with a 10^11^ Ω resistor in their feedback loop. Instrumental background signal contributions to each ion beam were removed by subtracting the signals obtained during 120 s of measurement prior to each analysis on the pure 0.28 M HNO_3_–0.01 M HF solution that was used for standard and sample preparation. Each sample and standard measurement comprises 100 ten-second-long integrations of the ion beam intensities. All gravimetric additions were done on a Mettler Toledo XP56 scale (10^–6^ g scale interval). In detail, four gravimetric mixtures of the ^93^Mo master solution with different amounts of the Mo standard solution and approximately the same amount of a Zr standard solution as well as one Mo standard-free mixture of the ^93^Mo master solution and the Zr standard solution were prepared. Note that if the same solution is used for all mixtures, the exact concentration of the Zr standard solution is irrelevant for the results of the standard addition with an internal standard.

The content of a stable isotope of Mo in the master solution (here: interference-free ^95^Mo and ^97^Mo) is then calculated from the linear change in.

- “Standard addition with internal standard”: the ^95^Mo and ^97^Mo signal intensity referenced (i.e., “divided by”) to the ^90^Zr or ^91^Zr ion beam intensity and multiplied by the weight ratio of the added internal standard and the ^93^Mo master solution in the mixture (see^22^ for general details and formulae) or
- “Reverse isotope dilution”: the measured ratios ^95^Mo/^93^Mo and ^97^Mo/^93^Mo of the mixture compared to the respective value of the Mo standard-free mixture (see^23^ for general details and formulae).

with the gravimetrically defined weight ratio of the added amount of the Mo standard and the master solution in the mixture.

Final concentration uncertainties are derived by Monte-Carlo propagation and include:

(i) the certified concentration uncertainty (*k* = 1) of the Mo reference solutions,
(ii) weighing uncertainties as one standard deviation obtained from repeatedly (*N* = 10) weighing each dilution and addition step during and before preparation of all mixtures containing the master solution,
(iii) the uncertainties of repeated (*N* = 5) mass spectrometric analyses of each mixtures containing the master solution as one standard deviation,
(iv) and the overall scatter in concentration results from combining all abovementioned uncertainties in
  - Monte-Carlo models of repeated linear regressions (*N* = 1 × 10^6^) for standard addition calculations and
  - Monte-Carlo models (*N* = 1 × 10^6^) of inverse isotope dilution.

### Liquid scintillation counting

The liquid scintillation (LS) measurements were carried out in two custom-built TDCR (i.e., triple-to-double coincidence ratio) counters at PTB. The first system is referred to as M27^24^ and it is equipped with a MAC3 coincidence unit^25^. The second TDCR counter, M29, makes use of an FPGA-based coincidence module referred to as the “4KAM”^26^. In addition, two commercial LS counters were used to apply the CIEMAT/NIST efficiency tracing (CNET) method: a Wallac 1414 Guardian™ liquid scintillation spectrometer and a TriCarb2800 TR from PerkinElmer. For details see Supplementary materials.

Two LS sample series were prepared. The first series was made using 20 mL glass vials, while 20 mL “low diffusion” polyethylene vials (with a “PTFE^®^-type coating on the inside surface”) were used for the second series. It has to be noted that all LS samples including the ^3^H samples (see below) were prepared from the same Ultima Gold (UG) batch. Small amounts of nitromethane were added to some of the samples in glass vials in order to reduce the counting efficiency by means of chemical quenching. The calibration curves, i.e. the counting efficiency of ^3^H as a function of the quenching indicator (called *SQP*(*E)* in case of the Wallac and *tSIE* in case of the TriCarb, respectively), were measured with the aid of a PTB standard solution of ^3^H. The LS samples containing ^3^H have the same sample composition and geometry as the ^93^Mo LS samples. All weighing procedures were carried out using a Mettler Toledo XP26, which is calibrated once a year ensuring traceability to the German national mass standard. LS spectra as well as an estimation of potential adsorption effects are given in the Supplementary materials.

## Results

The half-life of ^93^Mo was determined through the following steps:

1. Preparation of the ^93^Mo master solution free of radioactive impurities and isobaric interferences.
2. Determination of the ^93^Mo content by Multiple-Collector Inductively Coupled Plasma Mass Spectrometry (MC-ICP-MS).
  The sample should be free of isobars and molecules that could lead to interferences in the ICP-MS measurement.
3. Activity measurement of ^93^Mo by Liquid Scintillation (LS) counting with the following basic conditions to be fulfilled:
  The sample must not contain significant radioactive impurities influencing the activity determination and should be chemically stable over the period of measurement.

The uncertainties of the presented values in the manuscript are given for *k* = 1 if not stated otherwise.

### Production of ^93^Mo master solution

The element niobium is monoisotopic with mass 93. Therefore, the decontamination factor from the macro amount of Nb needed to be at least 1 × 10^12^ to avoid isobaric interference with ^93^Mo to make reliable MC-ICP-MS measurements possible. The achieved overall decontamination factor from niobium for the final purified ^93^Mo sample is larger than 1.6 × 10^14^.

The determined decontamination factors from Nb in different separation steps are presented in Table 1.

**Table 1:** Decontamination factors of ^93^Mo from Nb for each separation step.

Separation step	Decontamination factor from Nb		
	Disc nr. 1	Disc nr. 2	Disc nr. 3
TBP resin	1.20 × 10^5^	1.65 × 10^4^	5.52 × 10^3^
Alumina	1.20 × 10^5^	7.28 × 10^4^	1.20 × 10^6^
	Merged samples		
TBP resin	1.33 × 10^5^		
	Overall decontamination factor from Nb		
	1.6 × 10^14^		

Taking the mass of the niobium disc material that was used (1.8 g, corresponding to 1.65·10^22^ atoms) for molybdenum extraction into account, the amount of stable Nb in the master solution has been reduced to 4 × 10^7^ atoms or less. This amount of Nb contributes to the overall budget of mass-93 nuclides by less than 10^–7^ and is thus insignificant. Before preparation of the final master solution, the sample was measured for 5 × 10^5^ s live-time on an n-type HPGe detector (26% relative efficiency, Canberra) for detection of potential γ-ray emitting radioactive impurities. No gamma emissions were found apart from the natural background. The absence of radioactive impurities was also confirmed by LS counting measurements. After the chemical separation, the sample was evaporated to dryness and subsequently dissolved in 0.28 mol/L HNO_3_–0.01 mol/L HF mixture creating the “^93^Mo master solution” for both MC-ICP-MS and LS counting.

### Mass spectrometry analysis

**Figure 3:**
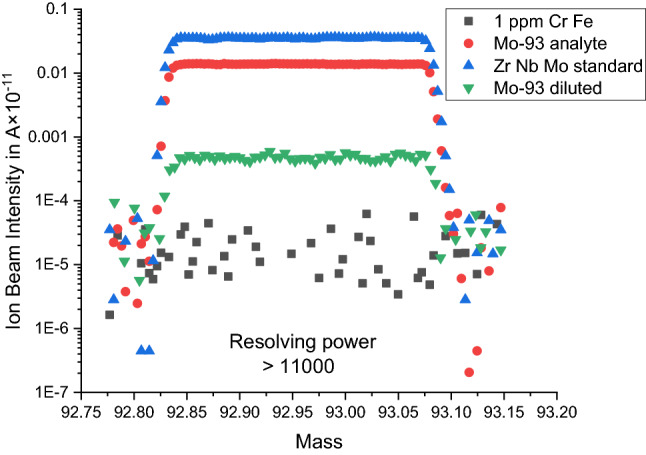
Signal profiles at high mass resolving power illustrating the lack of molecular interferences on mass 93 in the analyte. Molecular interferences whose mass difference to that of ^93^Mo is smaller than the mass range of the scan would show (e.g., from low to high mass: ^93^Mo-only, ^93^Mo^+^  + ^53^Cr^40^Ar^+^, ^53^Cr^40^Ar^+^-only) in the signal profile as prominent steps given the logarithmic scale. Further, note the lack of significant signal contributions from Metal Argides (MAr^+^) at metal (Cr, Fe) impurities on the ppm level.

A scan across the ^93^Mo signal peak utilizing the high mass resolution capabilities of the Nu Instruments Plasma 3 MC-ICP-MS indicates that the signal at mass 93 comes only from ^93^Mo atoms and potential molecular interferences can be excluded (Fig. 3).

No significant impurities in the sample analyte were found in a separate mass scan with the only discernable impurity being Fe at a concentration of ca. 1.5 ppm (see Supplementary materials). Note that this level of impurity does not produce detectable Metal-Argide signals (i.e., 40 mass units above the impurity’s mass) as attested by a scan across mass 93 on a 1 ppm Fe–Cr solution (Fig. 3). The recorded ion beam at mass 93 is thus free of bias from nuclides or molecules other than ^93^Mo.

**Figure 4:**
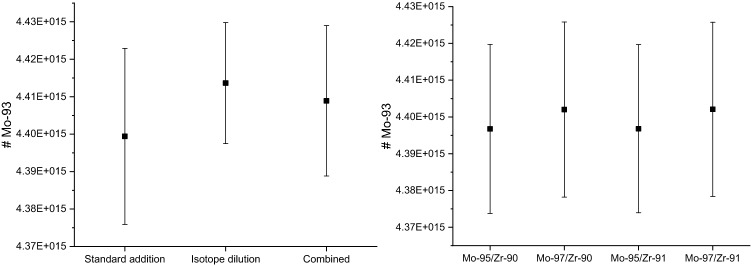
Left: ^93^Mo-amount results using either the isotope dilution or the standard addition method. Right: ^93^Mo-amount results from standard addition using either ^95^Mo or ^97^Mo, internally normalizing to either ^90^Zr or ^91^Zr and averaging results using a mass discrimination correction based on Mo and Zr.

The overall ^93^Mo-amount determination further is free from significant bias resulting from molecular interferences on the remaining involved masses as attested by the excellent agreement among ^93^Mo-results using either reverse isotope dilution or the standard addition method whose results agree within less than 0.16% (Fig. 4). Moreover, ^93^Mo-results from isotope dilution data based on ^93^Mo/^95^Mo and ^93^Mo/^97^Mo agree within less than 0.12%; results from standard addition using ^95^Mo or ^97^Mo and referencing to either ^91^Zr or ^90^Zr agree within less than 0.13% (Fig. 4).

The total number of ^93^Mo atoms per gram of the ^93^Mo master solution was determined to be 4.415(23) × 10^15^.

### Liquid scintillation counting

The measurements of the ^93^Mo LS samples and background samples yield experimental net count rates. In order to translate these into absolute activities, the corresponding counting efficiencies must be known. In this work the counting efficiencies were determined by means of the triple-to-double coincidence ratio (TDCR) method and the CIEMAT/NIST efficiency tracing (CNET) method^27^. These methods can be considered as being well established in radionuclide metrology. However, they have never been applied to ^93^Mo before. In the case of ^93^Mo, a major challenge is to compute the required LS counting efficiencies of the weak electron-capture (EC) decays. The computations included a stochastic atomic rearrangement model which proved its worth for several other EC radionuclides^28,29^. The calculations, however, require accurate nuclear and atomic data as input. In particular the fractional electron-capture probabilities must be well-known (see Supplementary material). The calculations also require knowledge on the decay probabilities of the two involved EC branches of ^93^Mo. The determination of these probabilities was achieved with a complex numerical procedure using long-term measurement data and is explained in the Supplementary materials, too. The probability *P*_EC,isomer_ is also required to estimate the growing contribution of ^93m^Nb to the overall counting rate.

**Figure 5:**
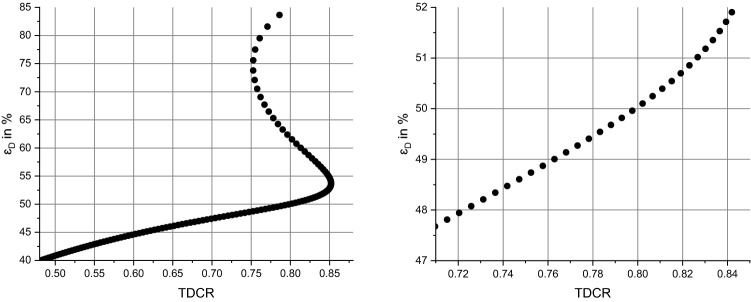
Computed double counting efficiency ε_D_ as a function of the TDCR parameter. The ambiguity is a well-known effect which occurs for several radionuclides with complex decay schemes (see, e.g.^27^). The right figure shows the same data in the ROI only.

Once, when the probabilities of the two EC branches are known, one can calculate the counting efficiencies for both methods. The ionization quenching function was computed using a Birks parameter *kB* = 0.0075 cm/MeV as described in^30^. Figure 5 shows the computed double coincidence counting efficiency *ε*_D_ as a function of the TDCR parameter. As for many complex decay schemes, the curve shows an ambiguity, i.e. parts where one TDCR value could lead to more than one value for *ε*_D_. In order to find the correct result, it is important to apply an efficiency variation (here realized by means of chemical quenching with nitromethane). When looking at the parts with high TDCR values, one can also identify a region where small changes of the TDCR parameter result in huge changes of the corresponding counting efficiency. This may lead to high uncertainties and a somewhat larger model dependence. Hence, all data with double counting efficiencies *ε*_D_ > 52.2% (in particular those with TDCR > 85%) were not used in the analysis of the activity concentration. This applies only to one measurement of a glass vial in the TDCR system M27 and all TDCR measurements with the PE vial.

**Figure 6:**
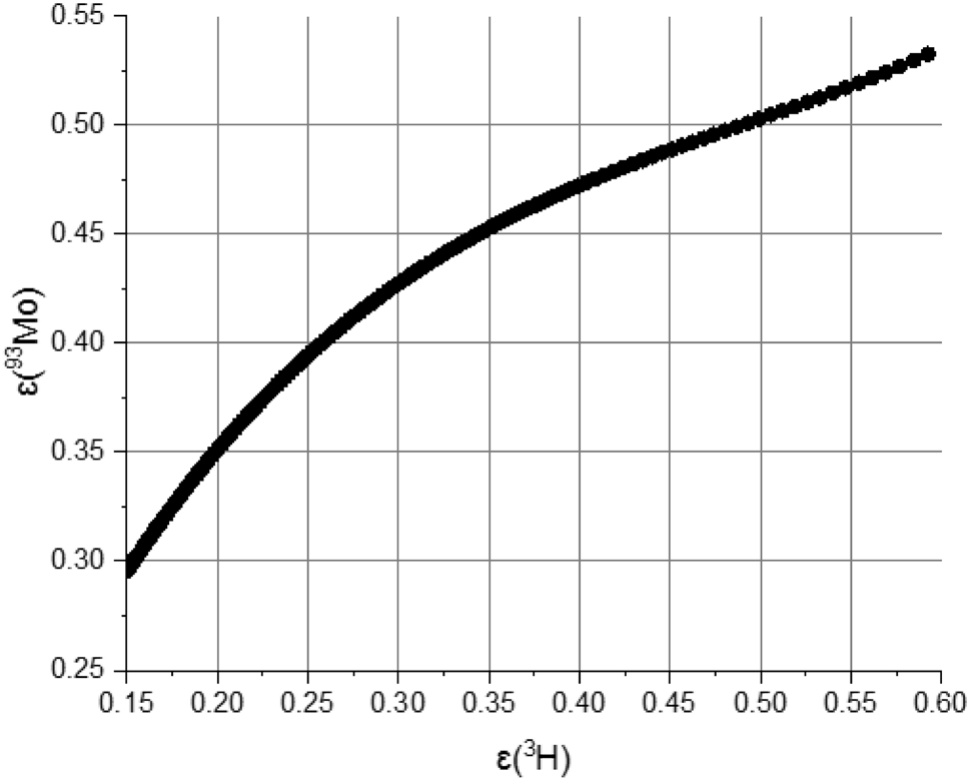
Computed ^93^Mo counting efficiency vs. ^3^H counting efficiency to apply the CNET method.

The efficiency curve for the CNET method is shown in Fig. 6. This curve does not show any ambiguity and no experimental data was discarded.

The counting efficiencies of ^93m^Nb were also calculated with the stochastic model but a detailed description is omitted here. It should, however, be noted, that the LS counting efficiency of ^93m^Nb is very high (e.g. the double counting efficiency in TDCR is about 99.7%) which is due to the fact that ^93m^Nb ejects mainly conversion electrons. Hence, both the variability of the counting efficiency and the related uncertainty are very small.

With the known value of *P*_EC,isomer_, it is straight forward to compute the counting efficiencies. All required corrections were applied (background subtraction, consideration of ingrowing ^93m^Nb, decay corrections). The quenching indicator *tSIE* measured in the TriCarb was found to be slightly biased. The bias was identified by comparing the ^93^Mo sample without nitromethane and the background sample which have basically the same composition. Such a bias was also observed for a few other radionuclides and may be explained by an overlap of the LS spectrum created by the radionuclide under study and the spectrum created due to the external ^133^Ba standard in the counter. After application of the correction, the TriCarb results (activity concentrations) are found to be about 0.45% larger than the uncorrected data. The Wallac data did not show any bias for the quenching indicator *SQP(E)*, and consequently, no correction was needed.

**Figure 7:**
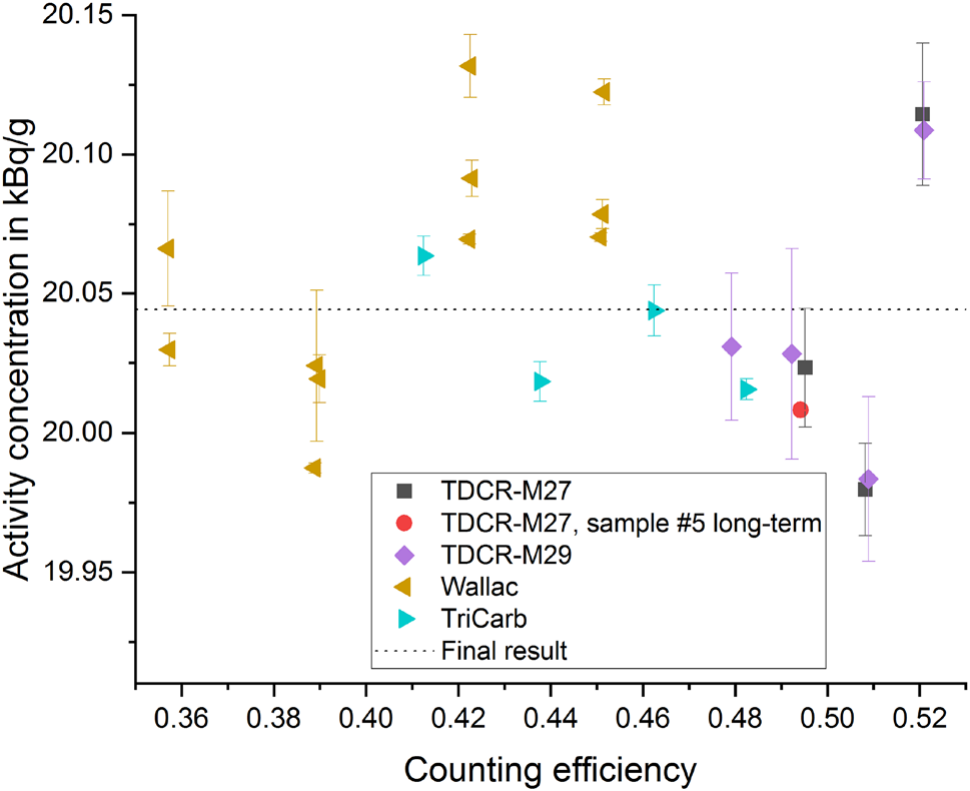
Activity concentration as obtained for the CNET and the TDCR method without data that were discarded. The uncertainty bars represent only a statistical component which was calculated as a standard deviation of the mean of several repetition measurements.

A figure that shows the determined activity concentration from all samples as determined using the TDCR and CNET method, respectively, is given in the Supplementary material. As expected, the TDCR data with (double) counting efficiency of more than 52% show a larger spread which can be explained from the TDCR efficiency curve (Fig. 5). Hence, these data are discarded. The overall spread of remaining results (Fig. 7) is much lower (standard deviation 0.22%).

The unweighted mean of the CNET results is *a*_CNET_ = 20.06(24) kBq/g with a relative standard uncertainty of 1.16%. The unweighted mean of the TDCR results is *a*_TDCR_ = 20.03(22) kBq/g with a relative standard uncertainty of 1.11%. The results agree very well. Since the uncertainties are similar too, the final result is obtained as the unweighted mean, which is *a* = 20.04(24) kBq/g. This result is also shown as dashed line in Fig. 7. The uncertainty was taken from the CNET result as a conservative approach. The probability of EC decay to the isomer state ^93m^Nb was determined to be *P*_EC,isomer_ = 95.7(16)% and, consequently, the probability for the EC branch to the ground state is 1 − *P*_EC,isomer_ = 4.3(16)%. Details for this determination are given in the Supplementary materials. All activity concentrations were stated for a reference date 1 July 2019, 0:00 CET. Full uncertainty budgets are shown in Supplementary material. For more detailed information on the uncertainty assessment in LS counting see^31^.

### The half-life of ^93^Mo and discussion

The half-life of ^93^Mo was calculated based on the measured number of ^93^Mo (${N}_{{93}_{\mathrm{Mo}}}$) atoms in the master solution and its measured specific activity (${A}_{{93}_{\mathrm{Mo}}}$) using the formula:

$$T_{1/2} = \frac{{\ln (2) \cdot N_{{93_{{{\text{Mo}}}} }} }}{{A_{{93_{{{\text{Mo}}}} }} }}$$

where ${N}_{{93}_{\mathrm{Mo}}}$ is the number of ^93^Mo atoms per gram of the master solution 4.415(23)∙10^15^ atoms/g and ${A}_{{93}_{\mathrm{Mo}}}$ its activity concentration 20.04(24) × 10^3^ Bq/g. For the half-life calculation one year is assumed to have 365.242198 days. Using these numbers, the half-life value of ^93^Mo was determined to be *T*_1/2_ = 4839(63) years*.* At the same time the probabilities of electron-capture decay to the radioactive isomer ^93m^Nb was re-determined yielding the value of 95.7(16)%. The obtained half-life value is compatible with the currently adopted value of 4000(800) years while the uncertainty has been decreased by a factor of 15. The new value for the probability of the decay to the radioactive isomer ^93m^Nb 95.7(16)% is by factor of 8 more precise than the currently accepted value of 88(12)%. The precise determination of the ^93^Mo half-life and its decay scheme provides now the possibility towards more accurate radionuclide inventory calculations together with better estimates of the radioactive dose emission from low and intermediate nuclear wastes over long time periods.

## Supplementary Information

Supplementary Information.
