# Supplementary material for: First direct determination of the 93Mo half-life
Source: Sci Rep. 2021 Oct 5;11:19788. doi: 10.1038/s41598-021-99253-5 (PMC8492754; doi:10.1038/s41598-021-99253-5)
Supplement: Supplementary file 1 — Supplementary Information. [file 41598_2021_99253_MOESM1_ESM.pdf]

**Supplementary material**

## Characterization of the niobium target

$^{93}$Mo was produced in a high purity niobium of 2.1 mm thickness that was used in a proton beam energy degrader at the PSI proton irradiation facility plate (Impurity content as given by the supplier, in ppm: Ta – 100; Si – 30; Fe - 15, W – 15; Mo – 10; Ti, Ni, Zr < 5.). The total accumulated proton charge on the degrader was estimated to be approximately 1 mAh. The production cross section for $^{93}$Nb(p,n)$^{93}$Mo reaction at 72 MeV proton energy is in the order of 10 mb leading to production of several kBq of $^{93}$Mo. The niobium degrader was stored for more than 5 years after its last use and had a dose rate of 10 µSv/h at contact when used for the present application. The degrader was investigated using an n-type HPGe detector (Canberra) and autoradiography to determine its radionuclide content and distribution. A picture of the degrader together with an autoradiographic image is given in Supplementary Supplementary Figure **1**.

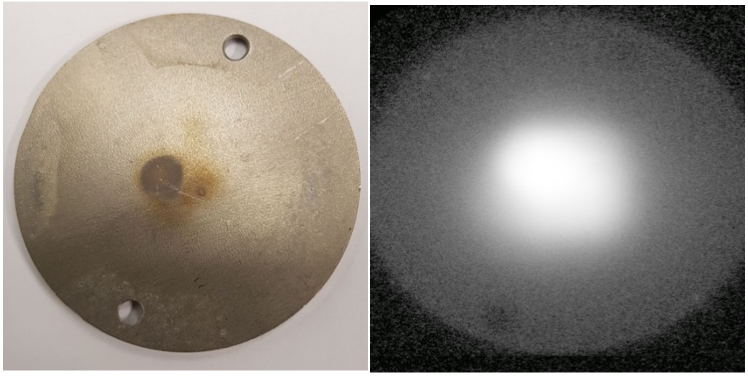

***Supplementary Figure 1:*** *Niobium disc after its use as proton energy degrader at the IP2 isotope production station at PSI (left). Autoradiographic image of the niobium disc after exposure (right).*

Subsequently, three circular samples with a diameter of 8 mm were mechanically cut out from the most active / tarnished locations of the disc using a blanking device. The punched out degrader samples were each weighed on the balance (Sartorius, 98648-018-34) and analyzed for the content of radioactive isotopes by means of γ and X-ray spectroscopy. X-ray measurements were performed on a low energy germanium (LEGe) detector (16 mm crystal diameter, 0.2 keV resolution at 123 keV line from the decay of $^{152}$Eu) provided by Canberra,

equipped with a carbon window. For the γ-ray measurements an n-type HPGe (Canberra) detector with relative efficiency of 26% with an aluminum window was used.

Both detectors were connected to a Lynx digital spectroscopic system. Collected spectra were evaluated with Genie2000 software. The γ and X-ray spectroscopic measurements of the retrieved Nb degrader revealed the presence of several long-lived isotopes in the sample. Apart from several niobium isotopes ($^{91}$Nb, $^{92}$Nb, $^{93m}$Nb and $^{94}$Nb), also small amounts of $^{22}$Na, $^{179}$Ta, $^{60}$Co and $^{54}$Mn were detected. As $^{93}$Mo emits the same energy of X-rays as $^{93m}$Nb (16.6 keV, 18.6 keV, 18.9 keV), these two radioisotopes cannot be distinguished in an X-ray spectrum. The recorded spectra are presented in Supplementary Supplementary Figure **2**.

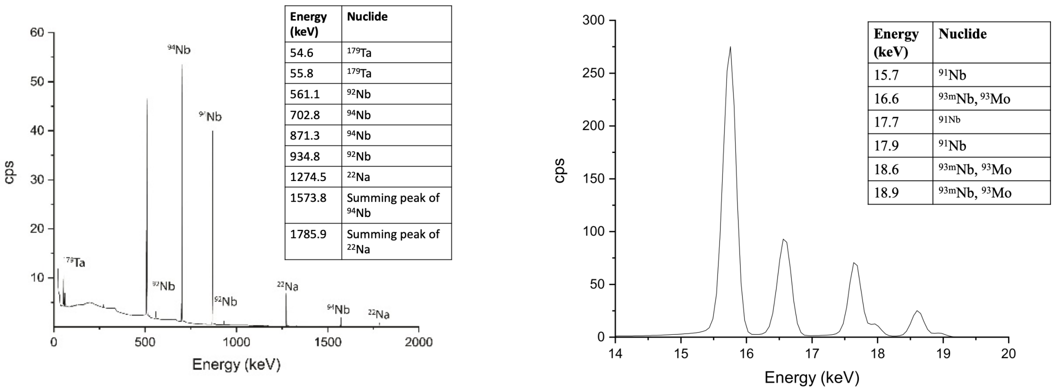


| Energy (keV) | Nuclide |
|---|---|
| 54.6 | $^{179}$Ta |
| 55.8 | $^{179}$Ta |
| 561.1 | $^{92}$Nb |
| 702.8 | $^{94}$Nb |
| 871.3 | $^{94}$Nb |
| 934.8 | $^{92}$Nb |
| 1274.5 | $^{22}$Na |
| 1573.8 | Summing peak of $^{94}$Nb |
| 1785.9 | Summing peak of $^{22}$Na |

| Energy (keV) | Nuclide |
|---|---|
| 15.7 | $^{91}$Nb |
| 16.6 | $^{93m}$Nb, $^{93}$Mo |
| 17.7 | $^{91}$Nb |
| 17.9 | $^{91}$Nb |
| 18.6 | $^{93m}$Nb, $^{93}$Mo |
| 18.9 | $^{93m}$Nb, $^{93}$Mo |

***Supplementary Figure 2:*** *Gamma (left) and X-ray (right) spectra of niobium degrader with identified main radioactive impurities.*

## Dissolution of the sample

The cut samples were transferred into 50 mL centrifuge vials and dissolved in 0.2 mL of 48% HF (Merck, Ultrapur) with 100 μL of 70% $HNO_3$ (J.T. Baker) at room temperature. Dissolutions were typically accomplished within one day at room temperature, i.e., 22(1) °C. The sample, dissolved in concentrated HF, was then mixed with 37% HCl (ACS grade, Sigma-Aldrich) and 18 MΩ·cm MilliQ water in order to get 50 mL of a 6.5 mol/L HCl - 0.1 mol/L HF solution.

## Separation procedure on TBP resin

For the separations on TBP resin a technique previously applied in solvent extraction [1] was modified for extraction chromatographic application described here. The solution with the dissolved parts of the niobium degrader was spiked with carrier free $^{95}$Nb tracer to determine

the decontamination factor from niobium. The sample was then transferred on the column with a length of 150 mm and internal diameter of 10 mm filled with 10 g of TBP resin (Triskem, France) with particle size of 50-150 μm preconditioned with the same acid composition using a peristaltic pump from Ismatec, Germany. After loading the sample, the resin was rinsed with 200 mL of the same acid mixture. The molybdenum fraction was finally eluted with 50 mL of 18 MΩ MilliQ water. A subsequent evaporation to dryness in the polyethylene vial occurred at 50 °C under a nitrogen gas flow on a heating plate overnight. For each separation on a TBP column, a fresh batch of resin was used.

The γ-spectroscopic measurements of the eluted fractions showed that $^{95}$Nb, $^{95}$Zr, $^{60}$Co, $^{54}$Mn and $^{22}$Na are not retained on the TBP resin in 6.5 mol/L HCl - 0.1 mol/L HF. By following the 16.6 keV Nb Kα line originating from the decay of $^{93}$Mo it was observed that Mo is retained quantitatively on the resin at the given conditions. Strong retention was also observed for tantalum and iron originating as impurities from the niobium degrader. During the elution phase with MilliQ water, the majority (>90%) of the adsorbed Mo was eluted together with Ta and a part of the iron contaminant. The decontamination factors from Nb and Zr after a separation on the TBP resin were observed not to be less than $5.5 \cdot 10^3$, limited by the detection limit of the HPGe detector. The purities of the used acids were ACS grade (HCl) and Ultrapure (HF) for the first separation step and Ultrapure NORMATOM (VWR) grade with certificate for all other separation steps. Certified niobium content in the Ultrapure NORMATOM grade was less than 0.01 ppt for both HCl and HF.

**Separation procedure on alumina substrate**

The second separation was performed using a slightly modified method reported by Osvath et al. [2], using alumina as chromatographic material. The dried sample from the separation on TBP resin was re-dissolved in 20 mL of 0.1 M HF and loaded on the alumina column with a length of 14.5 mm, internal diameter of 5 mm filled with 2 g of $Al_2O_3$ washed and preconditioned with 0.1 mol/L HF using a peristaltic pump. After loading of the sample, the alumina was washed with 40 mL of 0.1 mol/L HF and afterwards molybdenum was eluted with 20 mL of 20% $NH_4OH$ solution provided by VWR. For each separation on an alumina column, a fresh batch of $Al_2O_3$ was used.

Full retention of $^{93}$Mo, $^{95}$Nb, $^{95}$Zr and $^{179}$Ta was observed during the loading and washing stage with 0.1 mol/L HF. The elution process using 20% $NH_4OH$ is selective for Mo only, other trace elements remained on the column under these conditions. The lowest observed

decontamination factor from Nb during this procedure was $7 \cdot 10^4$, while typically, it was $1.2 \cdot 10^5$. The purity of the $NH_4OH$ solution was Ultrapure NORMATOM (VWR) grade. The certified niobium content in the Ultrapure NORMATOM grade was less than 0.01 ppt.

**Decontamination factors determination**

In order to trace the decontamination factors of Mo from Nb, as well as other radioactive impurities γ and X-ray spectroscopic measurements were used. As concentrations are proportional to the radioactivity of the radioactive isotope, here denoted as $A_L$ and $A_E$ activities were used for decontamination factors determinations.

The decontamination factor from Nb was determined by spiking the sample before each separation except the very last one with $^{95}Nb$ radiotracer dissolved in the same acid composition as the sample itself. The radiotracer $^{95}Nb$ was obtained from the in-house made $^{95}Zr/^{95}Nb$ generator. The generator has been based on the separation of $^{95}Zr/^{95}Nb$ on TEVA chromatographic resin (Triskem). Pure Zr metal foil was neutron irradiated at the SINQ facility of PSI in order to produce $^{95}Zr$. The neutron irradiated Zr foil was afterwards dissolved in 4 mol/L $HNO_3$, evaporated to dryness and re-dissolved in 7 mol/L HCl, 1 mol/L HF mixture. The mixture was applied on TEVA resin pre-conditioned with the same acid composition. In the given chemical environment Zr passes the column and is collected in the eluate while $^{95}Nb$ remains strongly absorbed on the resin. The elution (milking) of the $^{95}Nb$ from the resin was then executed with 6 mol/L HCl, 0.1 mol/L HF acid mixture. The obtained decontamination factor of the $^{95}Nb$ fraction from Zr impurities was in the order of $1 \cdot 10^4$ for all performed separations.

In order to avoid possible contamination of the $^{93}Mo$ sample with Nb as a possible impurity in Zr metal foil, the generator was milked and the first eluate was discarded. Following the 14 days in-growth period subsequent milking fractions were used as the source for $^{95}Nb$ radiotracer.

During the X-ray measurements the 16.6 keV Nb Kα-line originating from decay of $^{93}Mo$ and $^{93m}Nb$ in the sample together with 15.5 keV Zr Kα-line originating from the decay of $^{91}Nb$ were followed. After the first separation on TBP resin essentially all the signal at 16.6 keV line originated from $^{93}Mo$, making this energy convenient for tracing the behavior of molybdenum during the following separation steps. Thanks to high resolution of the LEGe detector the behavior of $^{179}Ta$ could be followed via its X-ray emissions with energies of 54.6 keV and 55.78 keV.

Gamma spectroscopic measurements were performed by following the 765.8 keV line from decay of $^{95}Nb$ ($T_{1/2}$=34.991(6) d) and 756.8 keV line from decay of $^{95}Zr$ ($T_{1/2}$=64.032(6) d). Due to the <5 ppm zirconium impurity in the niobium degrader, the decontamination from zirconium was followed during the first TBP separation. This was done in order to avoid the interference from $^{93}Zr$ in the molybdenum fraction formed by $^{93}Nb(n,p)^{93}Zr$ or $^{92}Zr(n,\gamma)^{93}Zr$ reactions in the irradiated degrader. The decontamination from Zr was monitored via spiking the solution with $^{95}Zr$ together with $^{95}Nb$. All measurements were performed with the same sample geometry before and after the separation to enable the calculation of the Nb and Zr decontamination factors. Decontamination factors for $^{60}Co$, $^{54}Mn$ and $^{22}Na$ were determined from the γ-ray measurement of the original niobium disc and measurement of the sample before the final separation. The differences in the full efficiency peak due to the changes in geometry and density of the sample were quantified by calculation utilizing the EFFTRAN code [3]. The determined overall decontamination factors for radioactive impurities other than Nb isotopes are presented in Supplementary Table 1.

**Supplementary Table 1.** The overall decontamination factors for radioactive impurities in the sample.

| **Impurity** | **Decontamination factor** |
|---|---|
| $^{93(95)}Zr$ | $>1.7\cdot10^{9}$ |
| $^{179}Ta$ | $>2.1\cdot10^{7}$ |
| $^{22}Na$ | $>3.0\cdot10^{9}$ |
| $^{60}Co$ | $>1.0\cdot10^{5}$ |
| $^{54}Mn$ | $>1.0\cdot10^{5}$ |

One more separation on TBP resin was performed before creating the "$^{93}Mo$ master solution". This separation was performed less than 48 hours prior starting the LS measurements in order to decontaminate the sample from $^{93m}Nb$ daughter formed by the decay of $^{93}Mo$. This separation was performed without radioactive tracers to prevent from possible contamination by $^{95}Nb$. As the last separation was performed without radioactive tracers, the decontamination factor for this separation cannot be quantified and thus, this decontamination step was not taken into the account when calculating the final decontamination factor. Since a conservative separation factor of $1\cdot10^{3}$ can be surely assumed based on robustness and reproducibility of the method,

an overall decontamination factor of at least two to three orders of magnitude higher can be assumed.

## Details on liquid scintillation counting

## LS spectra

Supplementary Figure 3 (left) shows a measured liquid scintillation spectrum of a $^{93}$Mo LS sample in a PE vial obtained with the Wallac counter which has a logarithmic amplification. A corresponding background spectrum has been subtracted. The spectrum does not contain significant entries above channel 300. Hence, potential significant impurities of high energy beta emitters (e.g. $^{94}$Nb with $E_{\max}$=471.7 keV, $^{95}$Nb with $E_{\max}$=159.8 keV) can be ruled out. The spectrum is compared with a similar spectrum obtained when measuring a $^{93m}$Nb LS sample (right) in the same counter. A slightly different sample composition was used in the case of $^{93m}$Nb (15 mL UG AB instead of UG). The $^{93m}$Nb spectrum shows some entries at high channel numbers which are assigned to a known impurity of $^{94}$Nb.

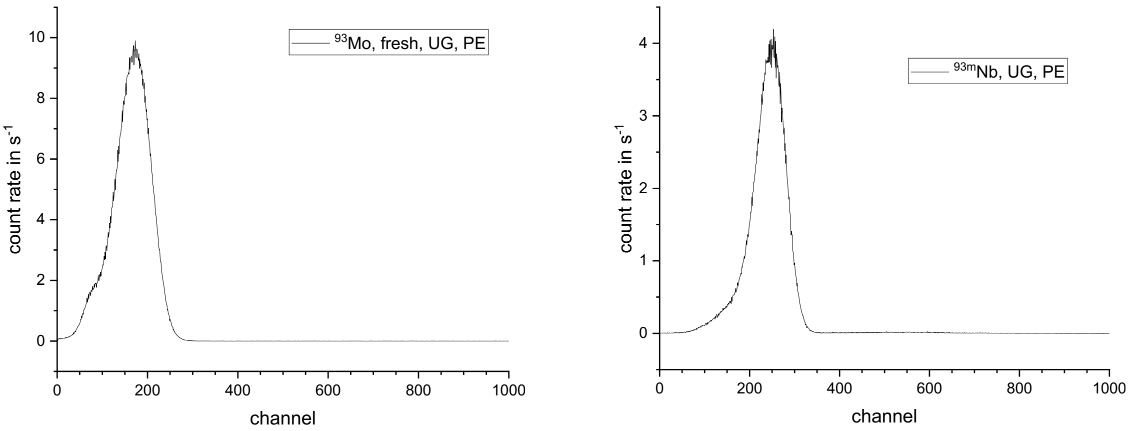


**Supplementary Figure 3**: *LS spectra of $^{93}$Mo (left) and $^{93m}$Nb (right) recorded with a Wallac 1414 LS counter. Corresponding background spectra have been subtracted. The $^{93}$Mo spectrum was taken about 5 days after the chemical separation, hence, the calculated $^{93m}$Nb portion amounts to be about $A(^{93m}Nb)/ A(^{93}Mo)\approx 5.6\cdot 10^{-4}$ only. Thus, the spectrum in denoted as "fresh".*

## Estimation of potential adsorption effect

After LS sample preparation, the original ampoule which was shipped from PSI to PTB was stored for 4 days in order to completely dry small remaining parts of the solution. The ampoule

was then weighed again and from the difference a total mass of solution of 1101.56 mg was determined. When subtracting the masses of all samples that were prepared, one can roughly estimate an upper limit of the activity that remained in the original ampoule (it is an upper limit because an unknown amount of the solution is discarded prior to drop deposition when preparing samples). This upper limit was estimated to be 270 Bq which corresponds to about 1.2% of the total activity which was provided by PSI. The ampoule was then rinsed with about 1 mL of distilled water which was then used to prepare another LS sample. This procedure was repeated three times in total. Finally, the empty ampoule was filled with UG before sealing it with an adhesive tape. The ampoule was then measured in the TriCarb counter using a PE adapter as a holder. A rough estimate of the total activity using the rinsing procedure and the empty ampoule yields a total activity of 138 Bq which is smaller than the above estimated upper limit. The activity determined from the measurement of the original ampoule filled with UG was found to be 59 Bq which is a conservative upper limit since a counting efficiency of only 25% was assumed (considering a counting efficiency of about 50% for normal samples and assuming that $^{93}$Mo is sticking on the vial walls). This activity corresponds to about 0.26% of the total activity delivered by PSI and a corresponding uncertainty component is taken into account. This can be considered as very conservative.

**Calculation of fractional EC probabilities**

The LS efficiency calculations require the fractional EC probabilities. The dominant EC decay leading to the $^{93m}$Nb isomer is of an 1$^{st}$ forbidden unique nature, and its fractional EC probabilities were calculated with the new BetaShape program Version: 2.0 (15/10/2019) which was recently improved and found to be reliable for the computation of allowed and unique forbidden beta transitions and EC decays [4]. For the calculation a transition energy of $Q^{+}$= 375.0 (15) keV was used which was taken from the AME2016 evaluation [5]. The EC probabilities were calculated to be $P_{K}$ = 0.8438(5), $P_{L1}$ = 0.11516(21), $P_{L2}$ = 0.001741(8), $P_{L3}$ = 0.00885(11) with the uncertainties from the original code stated in parenthesis. Since the stochastic model used in this work does not consider capture of electrons from shell $L_3$ a simplification was made, applying $P_{L2'} = P_{L2}+P_{L3}$ = 0.010591. A probability for EC from other shells (M and higher) is calculated by $P_{M+}$=1- $P_{K}$-$P_{L1}$-$P_{L2}$-$P_{L3}$= 0.030449. The EC decay to the ground state is of a 2$^{nd}$ forbidden non-unique nature. The EC probabilities were computed with the LOGFT program and found to be $P_{K}$ = 0.8631(1), $P_{L}$ = 0.11135(8). The probability $P_{L}$ was split to obtain $P_{L1}$ = 0.1097 and $P_{L2'}$ = 0.0017 following a formalism described in Browne and Firestone [6] using squared amplitudes of the bound-state electron radial wavefunctions.

Consequently, one obtains $P_{M+}$=0.0255. It should be noted that the computation of EC probabilities of the non-unique forbidden transition must be considered as an approximation since the calculation actually requires to take the nuclear structure into account.

**Details on the determination of the EC decay probability to the isomer** $P_{\mathrm{EC,isomer}}$

The probability for EC decay leading to $^{93m}$Nb was determined by means of long-term TDCR measurements and a complex analysis which will be explained in the following.
As a starting point, the following assumptions are made:

1. We assume that only 2 decay branches of $^{93}$Mo exist: one leading to the ground state of the stable $^{93}$Nb, the second leading to the isomer $^{93m}$Nb.
2. We assume that the purification was perfect, i.e. at time $t$=0 there was no $^{93m}$Nb and no other radioactive impurity.
3. We assume that we know the end-of-separation time ($t$=0) and that the duration of the overall separation procedure was short. The end-of-separation time is 25 June 2019 10:49 CET; the final separation took less than 1.5 h.
4. We assume that we know the half-lives of $^{93}$Mo and $^{93m}$Nb.

The TDCR measurements yield the net counting rate of triple coincidences $R_T(t)$ and the net counting rate of double (logical sum) coincidences $R_D(t)$. In both cases, the corresponding background counting rates were experimentally determined and subtracted from the total counting rates.
In the following the subscript "EC,gs" denotes the branch to the ground state of $^{93}$Nb while "EC,isomer" denotes the branch to $^{93m}$Nb.
The double counting rate is the sum of three components, i.e.

$$R_{\mathrm{D}} = R_{\mathrm{D,EC,isomer}} + R_{\mathrm{D,EC,gs}} + R_{\mathrm{D,\,Nb\text{-}93m}} \tag{1}$$

and similar for triple coincidences

$$R_{\mathrm{T}} = R_{\mathrm{T,EC,isomer}} + R_{\mathrm{T,EC,gs}} + R_{\mathrm{T,\,Nb\text{-}93m}} \tag{2}$$

We can also write

$$R_{\mathrm{D}} = A_{\mathrm{Mo\text{-}93}} \cdot \left( P_{\mathrm{EC,isomer}} \cdot \varepsilon_{\mathrm{D,EC,isomer}} + \left(1 - P_{\mathrm{EC,isomer}}\right) \cdot \varepsilon_{\mathrm{D,EC,gs}} \right) + A_{\mathrm{Nb\text{-}93m}} \cdot \varepsilon_{\mathrm{D,Nb\text{-}93m}} \tag{3}$$

and

$$R_{\mathrm{T}} = A_{\mathrm{Mo\text{-}93}} \cdot \left( P_{\mathrm{EC,isomer}} \cdot \varepsilon_{\mathrm{T,EC,isomer}} + \left(1 - P_{\mathrm{EC,isomer}}\right) \cdot \varepsilon_{\mathrm{T,EC,gs}} \right) + A_{\mathrm{Nb\text{-}93m}} \cdot \varepsilon_{\mathrm{T,Nb\text{-}93m}} \tag{4}$$

where $\varepsilon_{\mathrm{D,EC,isomer}}$, $\varepsilon_{\mathrm{D,EC,gs}}$ and $\varepsilon_{\mathrm{D,Nb\text{-}93m}}$ are the double counting efficiencies of the EC branch to the isomer, the EC branch to the ground state, and $^{93m}$Nb, respectively. The corresponding triple counting efficiencies are denoted as $\varepsilon_{\mathrm{T,EC,isomer}}$, $\varepsilon_{\mathrm{T,EC,gs}}$ and $\varepsilon_{\mathrm{T,Nb\text{-}93m}}$.

The activities $A_{\mathrm{Mo\text{-}93}}$ and $A_{\mathrm{Nb\text{-}93m}}$ in Equations (3) and (4) are time dependent, i.e.

$$A_{\mathrm{Mo\text{-}93}} = A_{\mathrm{Mo\text{-}93}}(t) = A_{\mathrm{Mo\text{-}93,0}} \cdot \mathrm{e}^{-\lambda_{\mathrm{Mo\text{-}93}} t} \tag{5}$$

with $A_{\mathrm{Mo\text{-}93,0}} = A_{\mathrm{Mo\text{-}93}}(t=0)$ and

$$A_{\mathrm{Nb\text{-}93m}} = A_{\mathrm{Nb\text{-}93m}}(t) = P_{\mathrm{EC,isomer}} \frac{\lambda_{\mathrm{Nb\text{-}93m}}}{\lambda_{\mathrm{Nb\text{-}93m}} - \lambda_{\mathrm{Mo\text{-}93}}} A_{\mathrm{Mo\text{-}93,0}} \left( \mathrm{e}^{-\lambda_{\mathrm{Mo\text{-}93}} t} - \mathrm{e}^{-\lambda_{\mathrm{Nb\text{-}93m}} t} \right) \tag{6}$$

Equations (3) and (4) contain the counting efficiencies. All 6 counting efficiencies can be calculated as a function of the free parameter. The free parameter makes it possible to link the results. We define

$$TDCR_{\mathrm{Mo\text{-}93,isomer}} = \frac{R_{\mathrm{T,EC,isomer}}}{R_{\mathrm{D,EC,isomer}}} \tag{7}$$

We can then calculate 6 efficiency curves, namely $\varepsilon_{\mathrm{D,EC,isomer}}(TDCR_{\mathrm{Mo\text{-}93,isomer}})$, $\varepsilon_{\mathrm{T,EC,isomer}}(TDCR_{\mathrm{Mo\text{-}93,isomer}})$, $\varepsilon_{\mathrm{D,EC,gs}}(TDCR_{\mathrm{Mo\text{-}93,isomer}})$, $\varepsilon_{\mathrm{T,EC,gs}}(TDCR_{\mathrm{Mo\text{-}93,isomer}})$, $\varepsilon_{\mathrm{D,Nb\text{-}93m}}(TDCR_{\mathrm{Mo\text{-}93,isomer}})$ and $\varepsilon_{\mathrm{T,Nb\text{-}93m}}(TDCR_{\mathrm{Mo\text{-}93,isomer}})$.

A first approximation to obtain $TDCR_{\mathrm{Mo\text{-}93,isomer}}$ is to use the experimentally determined TDCR value. This approximation is justified, since the activity of $^{93m}$Nb is low at the beginning and the efficiencies of the two EC branches are very similar.

We can then recalculate the ratio applying

$$TDCR_{\mathrm{Mo\text{-}93,isomer}} = \frac{R_{\mathrm{T,net,experimental}} - A_{\mathrm{Nb\text{-}93m}} \cdot \varepsilon_{\mathrm{T,Nb\text{-}93m}} - A_{\mathrm{Mo\text{-}93}} \cdot (1 - P_{\mathrm{EC,isomer}}) \cdot \varepsilon_{\mathrm{T,EC,gs}}}{R_{\mathrm{D,EC,isomer}} - A_{\mathrm{Nb\text{-}93m}} \cdot \varepsilon_{\mathrm{D,Nb\text{-}93m}} - A_{\mathrm{Mo\text{-}93}} \cdot (1 - P_{\mathrm{EC,isomer}}) \cdot \varepsilon_{\mathrm{D,EC,gs}}} \tag{8}$$

and apply an iteration, i.e. use the new result from Eq. 8 to recalculate the 6 efficiencies which are again used for Eq. 8.

This procedure allows us to compute the counting efficiencies with better precision. The outcome is, however, correlated with the outcome of a second procedure to determine $P_{\mathrm{EC,isomer}}$ and $A_{\mathrm{Mo\text{-}93,0}}$. This procedure will be described in the following.

The $^{93m}$Nb activity can also calculated applying

$$A_{\mathrm{Nb\text{-}93m,\ from}\ R_{\mathrm{D}}} = \frac{R_{\mathrm{D,net,experimental}} - A_{\mathrm{Mo\text{-}93}} \cdot \varepsilon_{\mathrm{D,\ Mo\text{-}93}}}{\varepsilon_{\mathrm{D,Nb\text{-}93m}}}, \tag{9}$$

where

$$\varepsilon_{\text{D, Mo-93}} = \varepsilon_{\text{D, EC, isomer}} \cdot P_{\text{EC,isomer}} + \varepsilon_{\text{D, EC, gs}} \cdot (1 - P_{\text{EC,isomer}}). \tag{10}$$

(Note: A similar methodology can be carried out using triple coincidences).

The two possibilities to calculate the $^{93m}$Nb activity from Eqs. 6 and 9 can be used for a minimization procedure:

$$\sum_{i=1}^{N} \left( \frac{A_{\text{Nb-93m, from } R_{\text{D}},i} - A_{\text{Nb-93m}}(t_i)}{A_{\text{Nb-93m}}(t_i)} \cdot w_i \right)^2 \rightarrow Minimum \tag{11}$$

using $P_{\text{EC,isomer}}$ and $A_{\text{Mo-93,0}}$ as variable parameters. The variable $i$ in Eq. 11 represents a single measurement and the sum is applied to all $N$ measurements. The weights were defined as $w_i = \sqrt{A_{\text{Nb-93m}}(t)\varepsilon_{\text{D,Nb-93m}} \cdot t_{\text{meas}}}$ with $t_{\text{meas}}$ being the duration of measurement (3600 s).

The procedure described above can easily be realized using the Excel-Solver tool which also makes a combination with the above-mentioned iteration process possible. The procedure requires initial estimates for the parameters $P_{\text{EC,isomer}}$ and $A_{\text{Mo-93,0}}$. An initial value for $A_{\text{Mo-93,0}}$ can easily be obtained since the $^{93m}$Nb activity is low at the beginning and the $^{93}$Mo counting efficiencies can be calculated from the free parameter model with rather low dependence on $P_{\text{EC,isomer}}$ (see discussion below). For $P_{\text{EC,isomer}}$ itself we can use any start value as long as it fulfils $0 < P_{\text{EC,isomer}} \leq 1$. This range is also defined as the only constraint for the minimization procedure.

**Discussion**

The described methodology works very well, and one might wonder "why?". The minimization procedure is based on two different ways to calculate the time-dependent $^{93m}$Nb activity. In the first way (Eq. 6) the probability $P_{\text{EC,isomer}}$ occurs as a factor (high correlation). The probability $P_{\text{EC,isomer}}$ is also required in Eq. 9, but its influence is very low. The counting efficiency $\varepsilon_{\text{D,Nb-93m}}$ is very high (about 99.7%) and is not very sensitive to changes of $P_{\text{EC,isomer}}$ (or $TDCR_{\text{Mo-93,isomer}}$ ). The efficiency $\varepsilon_{\text{D, Mo-93}}$ in the numerator of Eq. 9 depends on $P_{\text{EC,isomer}}$ (see Eq. 10). This dependence is, however, very much reduced, due to the fact that the counting efficiencies for the two $^{93}$Mo EC branches are similar.

We start now with the analysis of the long-term measurement of sample #5. Supp. Fig. 4 shows the net counting rates for double (left) and triple (right) coincidences as a function of time since

separation. Both plots show a significant decrease during the first days. The reason for this effect is not known but might be due to the fact that the UG bottle was freshly opened. Hence, oxygen may cause quenching effects which in turn reduce the counting efficiency. This finding is confirmed by decreasing trends of the quench indicating parameters (*SQP*(*E*) and *tSIE*) which were observed in the commercial LS counters. Lower quench indicating parameters also correspond to lower counting efficiencies.

Due to the ingrowing $^{93m}$Nb one expects an increase of the counting rates with time. Indeed, the double counting rate increases (after the first few days), but a somewhat larger slope was expected. The triple counting rate was first found to be more or less stable and starts to increase later. This means that the TDCR parameter decreases with time (Supp. Fig. 5).

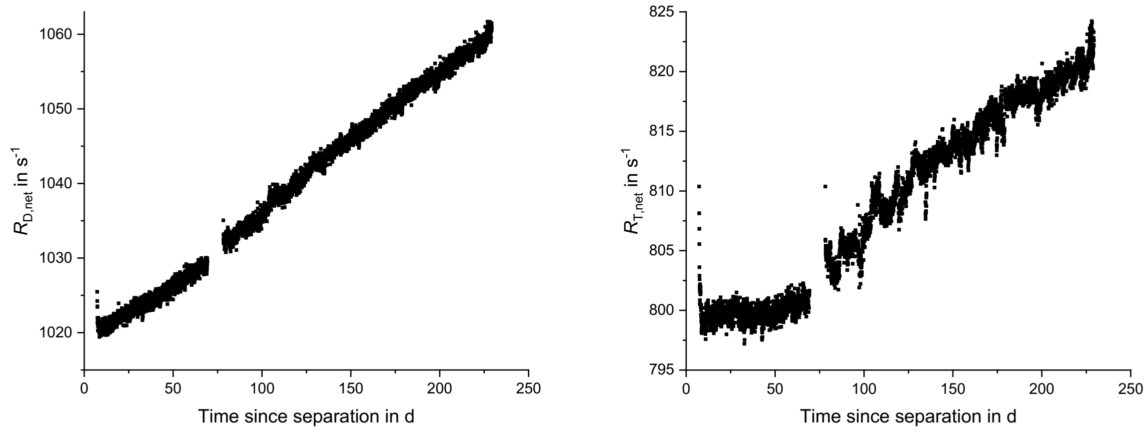


**Supplementary Figure 4**: *Net counting rates for double coincidences (left) and triple coincidences (right) as measured in TDCR-M27 using the LS sample #5 in a glass vial.*

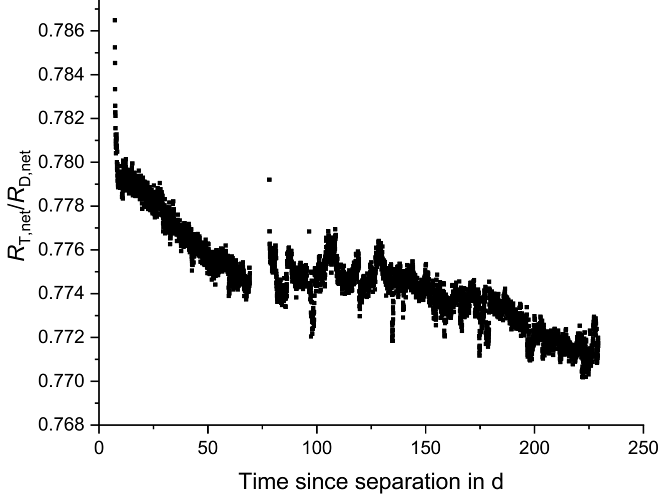

**Supplementary Figure 5**: *TDCR parameter as function of time since separation as determined in TDCR-M27 using the LS sample #5 in a glass vial.*

A decreasing TDCR parameter can be attributed to a decrease of the counting efficiency (at least when considering the ROI in the efficiency curve, see right side of Figure 5 in the main text). Hence, this significant trend is a strong indication of a related change (decrease) of the counting efficiency over time. This may also be due to oxygen that comes into the LS samples. One should note that such an effect, would be less pronounced when measuring radionuclides with higher overall counting efficiency (e.g. $^{14}C$). (Remark: For a stable sample, i.e. a constant free parameter, one would even expect an increasing TDCR value with time, due the ingrowth of $^{93m}Nb$).

Supp. Fig. 6 (left) shows the residuals $\frac{A_{\text{Nb-93m, from } R_D, i} - A_{\text{Nb-93m}}(t_i)}{A_{\text{Nb-93m}}(t_i)}$ as a function of time after application of the minimization procedure when taking all experimental data of sample #5 and the weights $w_i$ into account. A similar plot (right) is shown when applying the same procedure with the triple coincidences.

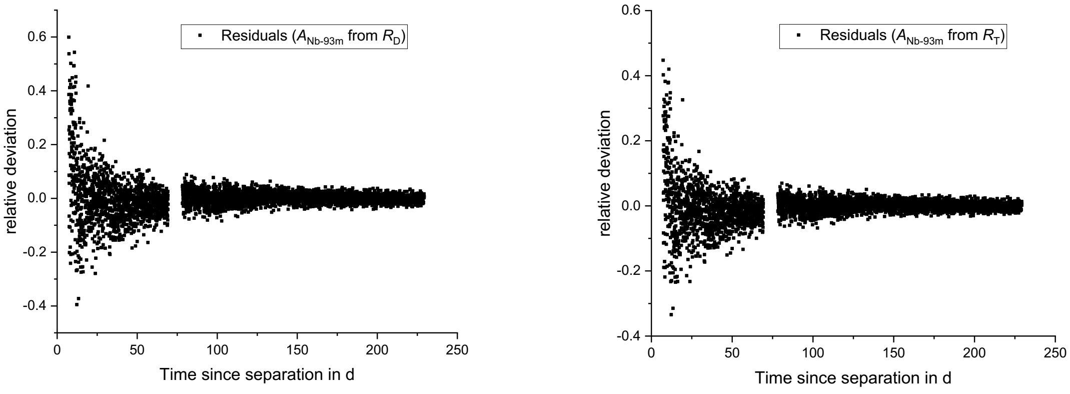


**Supplementary Figure 6**: *Residuals* $\frac{A_{\text{Nb-93m, from } R_D, i} - A_{\text{Nb-93m}}(t_i)}{A_{\text{Nb-93m}}(t_i)}$ *and* $\frac{A_{\text{Nb-93m, from } R_T, i} - A_{\text{Nb-93m}}(t_i)}{A_{\text{Nb-93m}}(t_i)}$ *as a function of time.*

When having a closer look to the gap in Supp. Fig. 6 between day 71 and day 80 one can identify a little step in the order of 0.025%. This might be due to a minor difference in the position of the vial after the interruption of a few days.

The minimization procedure as described above was first done with all measurement data using the weights. The result was found to be $P_{\text{EC,isomer}} = 95.7\%$. In order to check the stability of the

procedure and to estimate uncertainties, the fit was repeated with different conditions. For example, the $^{93m}$Nb half-life was varied within its uncertainty and the overall numerical procedure was repeated. The relative deviation of the new result for $P_{\mathrm{EC,isomer}}$ to the previous one was then taken as the relative uncertainty component. Similar changes were carried out for the $^{93}$Mo half-life (5000 y instead of 4839 y) and the computed counting efficiencies of $^{93}$Mo (varied by 1%). The algorithm was also repeated using only those experimental data taken 80 days after separation or later. Eventually, the algorithm was repeated with an unweighted minimization, i.e. $w_i$=1. All this helps to establish the uncertainty budget as shown in Supplementary Table 2.

**Supplementary Table 2**: Uncertainty budget for the determination of $P_{\mathrm{EC,isomer}}$.

| **Uncertainty component** | **Relative uncertainty** |
|---|---|
| Minimization algorithm (weighted vs. unweighted, selection of subset of data) | 0.61 % |
| $^{93m}$Nb half-life | 0.91 % |
| $^{93}$Mo half-life | 0.01 % |
| LS counting efficiency for $^{93}$Mo (model dependence, nuclear decay data) | 0.99 % |
| End of separation time, potential imperfect removal of $^{93m}$Nb | 0.68 % |
| **Total relative uncertainty** | **1.63%** |

As the final result, we obtain $P_{\mathrm{EC,isomer}} = (95.7 \pm 1.6)\%$. The probability for the EC branch to the ground state is then $1 - P_{\mathrm{EC,isomer}} = (4.3 \pm 1.6)\%$.

**Details on the activity determination**

The numerical procedures presented above give of course also access to the $^{93}$Mo activity. Supp. Fig. 7 shows the activity $A_0$ (i.e. corrected for decay) as a function of time.

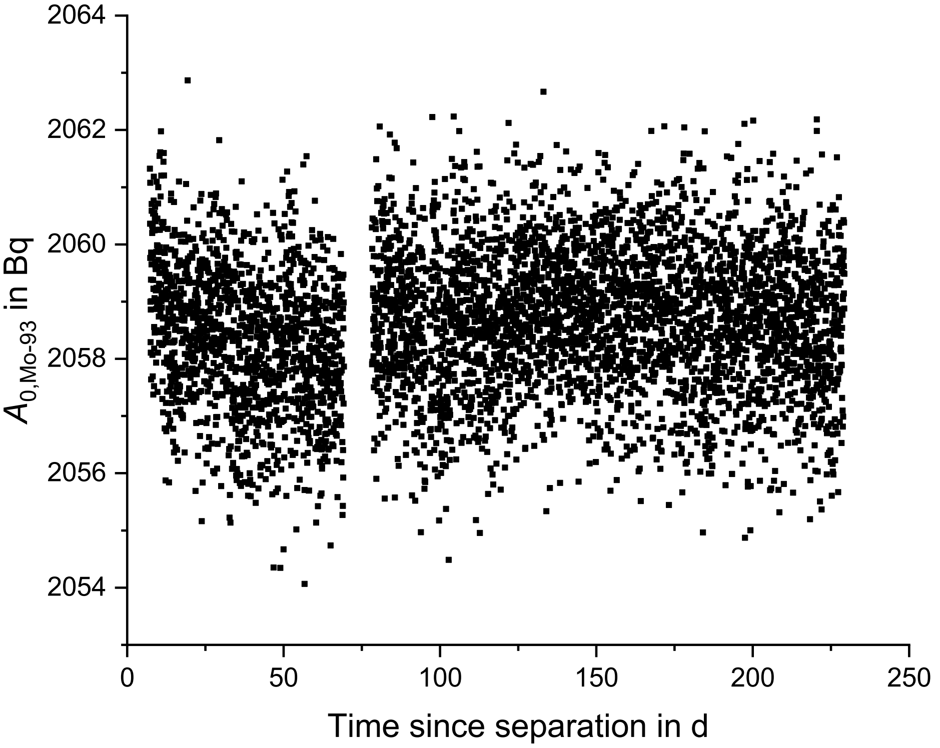


**Supplementary Figure 7**: *The $^{93}$Mo activity $A_0$ as a function of time since separation as obtained from the long-term measurement in TDCR-M27 of sample #5.*

The plot shows that the results are consistent. This is noteworthy since the variations in the counting rates and the TDCR are quite large (Supp. Figs. 4 and 5). The good stability seen in Supp. Fig. 7 indicates that the TDCR model applied here compensates the efficiency changes very well. It is to be noted that the overall counting efficiency for $^{93}$Mo is rather low (e.g. when compared to beta emitters with intermediate or high energies) and hence minor variations as seen in Supp. Fig. 7 were anticipated.

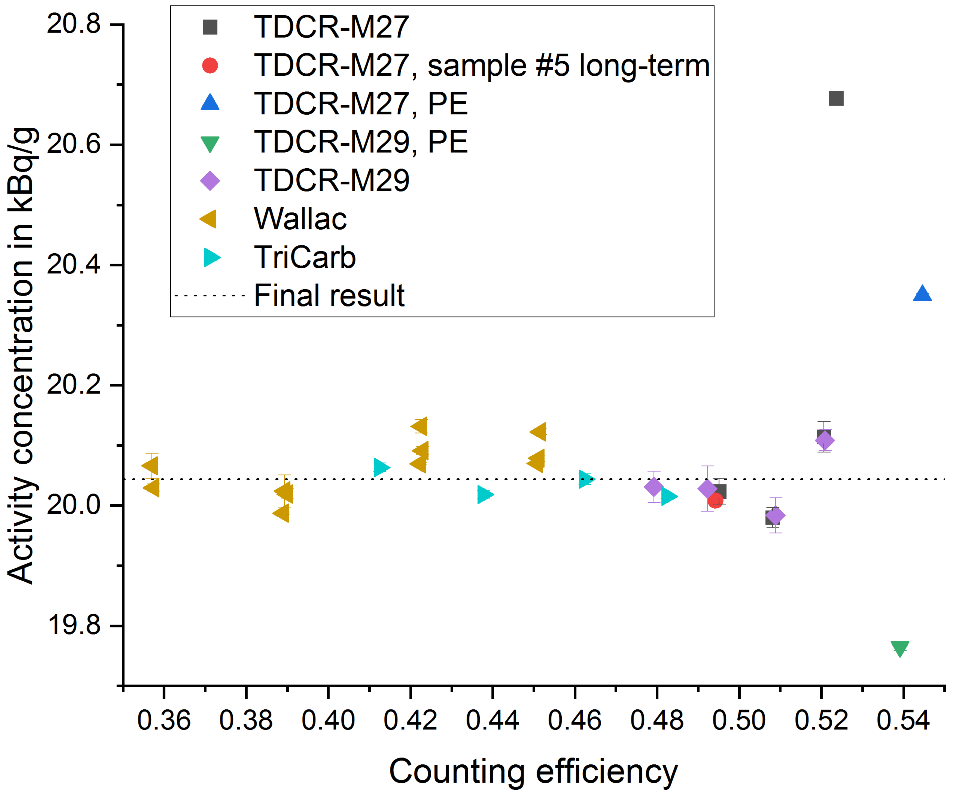


**Supplementary Figure 8**: *Activity concentration as obtained for the CNET and the TDCR method (all data). The uncertainty bars represent only a statistical component which was calculated as a standard deviation of the mean of several repetition measurements.*

The detailed uncertainty budgets for both activity determination methods are given in Supplementary Table 3. For more detailed information on the uncertainty assessment in LS counting see [7].

**Supplementary Table 3**: Standard uncertainty components assigned to the activity concentration *a* of a $^{93}$Mo solution measured by liquid scintillation counting.

| | **TDCR** | **CNET** |
|---|---|---|
| **Component** | ***u*(*a*)/*a* in %** | ***u*(*a*)/*a* in %** |
| Relative standard deviation | 0.24 | 0.20 |
| Weighing (LS samples) | 0.02 | 0.02 |
| Deadtime | 0.05 | 0.07 |
| Background | 0.07 | 0.05 |
| Adsorption (see text) | 0.26 | 0.26 |
| TDCR value | 0.22 | - |
| $^{3}$H standard as tracer | - | 0.44 |
| Radionuclide impurities (none detected) | 0.2 | 0.2 |
| Model and decay data (e.g. calculated LS counting efficiencies, fractional EC probabilities and probabilities of the EC branches, *kB* parameter etc.) | 1 | 1 |
| Decay correction | <0.01 | <0.01 |
| **Square root of the sum of quadratic components** | **1.11** | **1.16** |

**References**

[1] Silant'ev, A.I.; Iofa, B.Z.; Maklachkov, A.G. Separation of carrier-free molybdenum-93 from niobium targets, Soviet Radiochemistry, 1986. 27(5): p. 638-640

[2] Osváth, S., J. Qiao and X. Hou, Preparation of $^{93}$Mo solution using proton irradiated Nb. Journal of Radioanalytical and Nuclear Chemistry, 2019. 322(3): p. 1833-1839.

[3] Vidmar, T., EFFTRAN—A Monte Carlo efficiency transfer code for gamma-ray spectrometry. Nuclear Instruments and Methods in Physics Research Section A: Accelerators, Spectrometers, Detectors and Associated Equipment, 2005. 550(3): p. 603-608

[4] Mougeot, X., 2019. Towards high-precision calculation of electron capture decays. Appl. Radiat. Isot. 154, 108884.

[5] Wang, M., Audi, G., Kondev, F.G., Huang, W.J., Naimi, S., Xu, X., 2017. The AME2016 atomic mass evaluation. Chin. Phys. C 41, 03003

[6] Browne, E., Firestone, R.B.: Table of Radioactive Isotopes. Wiley, New York 1986.

[7] Kossert, K., Broda, R., Cassette, Ph., Ratel, G., Zimmerman, B., 2015. Uncertainty determination for activity measurements by means of the TDCR method and the CIEMAT/NIST efficiency tracing technique. Metrologia 52, S172-S190 (Special Issue on Uncertainty Evaluation in Radionuclide Metrology).
